# The Prevalence of Antimicrobial Resistant *Neisseria gonorrhoeae* in Papua New Guinea: A Systematic Review and Meta-Analysis

**DOI:** 10.3390/ijerph19031520

**Published:** 2022-01-28

**Authors:** Barne Willie, Emma L. Sweeney, Steven G. Badman, Mark Chatfield, Andrew J. Vallely, Angela Kelly-Hanku, David M. Whiley

**Affiliations:** 1 Centre for Clinical Research, Faculty of Medicine, The University of Queensland, Herston, QLD 4029, Australia; e.l.sweeney@uq.edu.au (E.L.S.); d.whiley@uq.edu.au (D.M.W.); 2 Papua New Guinea Institute of Medical Research, Goroka P.O. Box 60, Papua New Guinea; Avallely@kirby.unsw.edu.au (A.J.V.); a.kelly@unsw.edu.au (A.K.-H.); 3 Kirby Institute for Infection and Immunity, The University of New South Wales, Sydney, NSW 2052, Australia; sbadman@kirby.unsw.edu.au; 4 Faculty of Medicine, The University of Queensland, Brisbane, QLD 4072, Australia; m.chatfield@uq.edu.au; 5 Pathology Queensland Central Laboratory, Royal Brisbane and Women’s Hospital, Herston, QLD 4006, Australia

**Keywords:** *Neisseria gonorrhoea*, antimicrobial resistance, surveillance, Papua New Guinea

## Abstract

*Neisseria gonorrhoeae* antimicrobial resistance (NG AMR) has become an urgent concern globally. The World Health Organization, the United States of America Centers for Disease Control, and other regulators have called to improve resistance-testing methods to enhance NG AMR surveillance. NG AMR surveillance remains critical in informing treatment; unfortunately, this is often lacking in settings with limited resources, such as Papua New Guinea (PNG). We conducted a systematic review and a prevalence meta-analysis, and provided an overview of NG AMR in PNG. We showed the lack of NG AMR data in the last decade, and emphasized the need for NG AMR surveillance in PNG. Since NG AMR testing by the NG culture method is unreliable in PNG, we suggested using molecular tests to complement and enhance NG AMR surveillance.

## 1. Background

One of the most urgent threats to sexual health globally is the progressive development and spread of *Neisseria gonorrhoeae* antimicrobial resistance (NG AMR) [1,2]. NG can develop resistance in two main ways: through plasmid-mediated resistance (PMR), and, to a greater extent, through chromosomally mediated resistance (CMR), with the latter bolstered by frequent genetic material exchange with commensal *Neisseria* species [3]. Some public health commentators have predicted that NG infection may be becoming untreatable [1,2,4]. In response to this threat, the World Health Organization (WHO), the United States of America Centers for Disease Control and Prevention (USA-CDC), and other regulators have developed global action plans for improving the management of NG AMR [5]. Prominent within these documents are recommendations for developing rapid molecular assays to enhance the testing and surveillance of NG AMR [5,6].

The “gold standard” for NG AMR testing is the culture method [7]. However, it can be unreliable and particularly challenging to maintain in settings with limited resources [8,9]. For this reason, the WHO and others have called for developing molecular assays for NG AMR testing. Several in-house genotyping methods have been established and validated to test the presence of genes and gene mutations in NG that are associated with causing resistance towards the main class of antibiotics in treating it [2,10]. The advantages of NG AMR testing by molecular assays are that it does not require live bacteria for testing, and requires less stringent conditions for sample storage and transportation [8,9]. In addition, the samples can be self-collected by non-invasive techniques, such as urine or anorectal swabs, for molecular testing. The privacy and ease in sample collection for molecular testing thus increases sampling numbers, which improves surveillance of NG AMR by increasing sample size accuracy of prevalence estimates [10]. Though molecular testing methods are not as definitive as the culture method for determining NG AMR, they can effectively complement and supplement culture-based NG AMR surveillance.

The WHO recommended that national NG AMR surveillance be done at regular intervals to inform treatment. If observed resistance prevalence is above 5% for any antibiotics, a change in treatment is required [2,5]. Despite this, many low-to-middle-income countries (LMIC) with a high prevalence rate of NG infection cannot afford to do so. Typically, many LMIC lack local testing capacity, such as Papua New Guinea (PNG). Almost all countries in the WHO Western Pacific Region (WPR) and Southeast Asian Region (SEAR) have abolished the use of penicillin and ciprofloxacin treatment option due to the high prevalence of resistance, estimated at 70–100% for the two antibiotics [11]. Most are using cephalosporins (ceftriaxone and cefixime) and azithromycin drug combinations. However, cephalosporin (ceftriaxone) resistance prevalence is on the rise. Resistance in the range of 30–70% has been reported in Cambodia; 5–30% in China, Japan, and Taiwan; and 1–5% in Australia [12]. Elsewhere, the United States of America and Canada have reported a cephalosporin (ceftriaxone) resistance prevalence of 1–5%. Several European countries reported resistance prevalence to cephalosporins (cefixime) at 5–30% [12]. Interestingly, some countries in WPR and SEAR, such as China, Cambodia, Thailand, and Australia, reported an azithromycin resistance prevalence of 5–30% [12].

Papua New Guinea (PNG) has some of the highest prevalence of sexually transmitted infection (STI) in the WPR [13]. The recent gonorrhea prevalence estimate for PNG is 11% (95% CI: 4.5–16.1) [13]. However, this may still be an underestimation, as a relatively higher prevalence was previously reported, with a disproportionately higher prevalence among female sex workers (23–44% in 2010, and 15–33% in 2017) [14,15], men who have sex with men and transgender women (3–15% in 2017) [14], and people attending STI clinics (60–88% among males, and 13–41% among females in 2010) [15]. The high prevalence of NG infection alone in PNG is a concern, and more concerning is the lack of NG AMR data in the last decade. The existing NG AMR data is not sufficient and representative because of the small sample size from only several sampling sites across PNG. Therefore, up-to-date NG AMR data are crucially needed for PNG [16].

The standard treatment for gonorrhea in PNG is a single-dose cocktail of the following antibiotics: amoxicillin (penicillin), probenecid (uricosurics), augmentin (penicillinase/beta-lactamase inhibitor), and azithromycin (macrolide) [17]. Occasionally, doxycycline (tetracycline) or erythromycin (macrolide) are administered as a substitute for azithromycin [17], but this may change to a cephalosporin (cefixime and ceftriaxone) and azithromycin combination treatment for PNG [18]. However, there is insufficient NG AMR surveillance to inform such change [18]. In that light, our analysis aimed to provide an overview of the NG AMR trend, and discuss the need to explore molecular testing methods to supplement and complement the culture method for NG AMR surveillance in PNG.

## 2. Materials and Methods

### 2.1. Literature Search

The systematic review was conducted following the Preferred Reporting Items for Systematic Reviews and Meta-Analyses (PRISMA) method [19]. MEDLINE/PubMed [20], EMBASE [21], and Google Scholar were searched for papers published on NG AMR in PNG. Keywords search terms included “*Neisseria gonorrhoeae*” or “*Neisseria*” or “gonorrhoea” or “gonococcus” or “gonococcal” or “sexually transmitted infection” or “sexually transmitted disease” or “venereal disease” and “resistance” or “antibiotic resistance” or “antimicrobial resistance” and “Papua New Guinea”. The articles generated from the search were then screened following the set eligibility criteria outlined below for inclusion in the meta-analysis.

Inclusion criteria:

- Original study that investigated NG AMR in humans from PNG;
- Peer-reviewed articles published between January 1980 and October 2020. The cut-off year was set at 1980 and after was to ensure that the reported prevalence in the primary studies were generated using the Australia Gonococcal Surveillance Program (AGSP) and/or the WHO Gonococcal Antimicrobial Surveillance Program (GASP) methodology. These two programs use similar standardized methods for NG AMR testing and result interpretation;
- The total number of samples or isolates analyzed and the resistance prevalence described and stated. This was to ensure that we have the exact numerator (number of sample or isolates that were resistant to the antibiotic tested against) and denominator (total number of sample or isolates analyzed) to enable accurate proportion estimations in the meta-analysis; and
- Abstract and full text available in English.

### 2.2. Data Extraction and Analysis

Two independent reviewers (BW, ELS) screened the full articles. The data extracted from the papers were: article reference (following the Vancouver referencing style); country of study; year or period in which the study was conducted (year of sampling); year of publication; number of samples/isolates; and number of isolates that were successfully profiled for NG AMR for the following antibiotics of interest—penicillin (amoxicillin), tetracycline, macrolide (azithromycin), quinolone (ciprofloxacin), aminoglycoside (spectinomycin), and cephalosporins (ceftriaxone and or cefixime).

The data were collated and managed by removing duplicate articles in Microsoft Excel 365 (version 2020, Microsoft, Redmond, Washington, USA), and the Endnote referencing software (version 19, Clarivate Analytics, Philadelphia, USA) [22]. Prevalence meta-analysis statistical tests were performed using “metaprop” within “meta” (version 4.15-1) and “metafor” (version 2.4-0) CRAN packages in R statistical software, version 4.0.2 (RStudio 2020-06-22, Free Software Foundation Incorporated, Boston, Massachusetts, USA) [23]. The Random Effect Model (REM) within the software package was used for calculating the proportion estimates and the ninety-five percent confidence interval (95% CI) [24,25,26]. The REM estimator function was used because of the small number of primary studies and data points, and also because of high inconsistency in the primary studies’ reported prevalence and heterogeneity (I^2^ > 25%) [26].

## 3. Results

**Figure 1:**
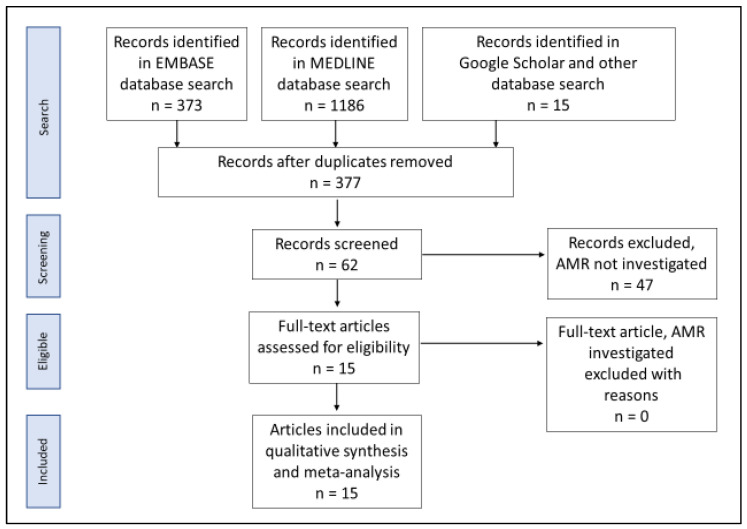
PRISMA [19] flow chart of article search, article inclusion and exclusion, and the number of studies included in this study-systematic review and meta-analysis.

A total of 1574 articles was generated following the literature search strategies mentioned above. All these papers were in English, and none in other languages. After removing duplicates, 377 articles remained. On further screening of the title and abstract of these 377 papers, 62 articles showed to report NG AMR in humans in PNG. Full-text articles of these 62 papers were accessed and screened following the inclusion criteria mentioned above. Out of the 62 articles, only 15 papers met the eligibility criteria and were included in the prevalence meta-analysis, see Figure 1. Forty-seven (47) papers were excluded because they were not original research studies, meaning they did not report on the case number and sample size. Of these 15 papers, the majority (80%) were generated through the WHO WPR GASP [27,28,29,30,31,32,33,34,35,36,37,38], and 3 (20%) were from other surveillance [16,39,40].

**Figure 2:**
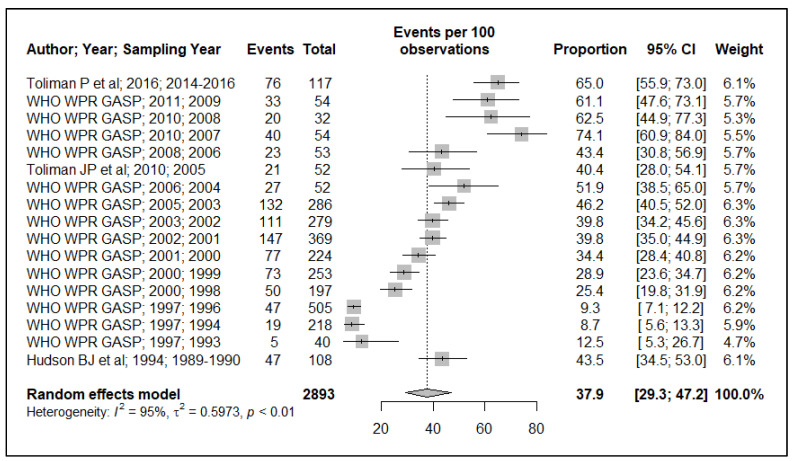
Penicillinase-producing *N. gonorrhoeae* positive strain prevalence estimate. Forest plots include the authors name, year of publication, year when samples were collected, number of cases reported, sample size employed, proportion, 95% CI, and sample weights. Diamond symbol at the bottom is the pooled estimate.

**Figure 3:**
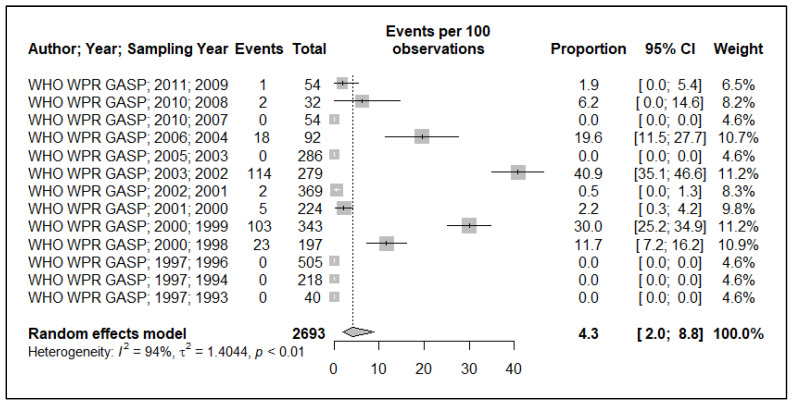
Chromosomally-mediated resistance to penicillin prevalence estimate.

**Figure 4:**
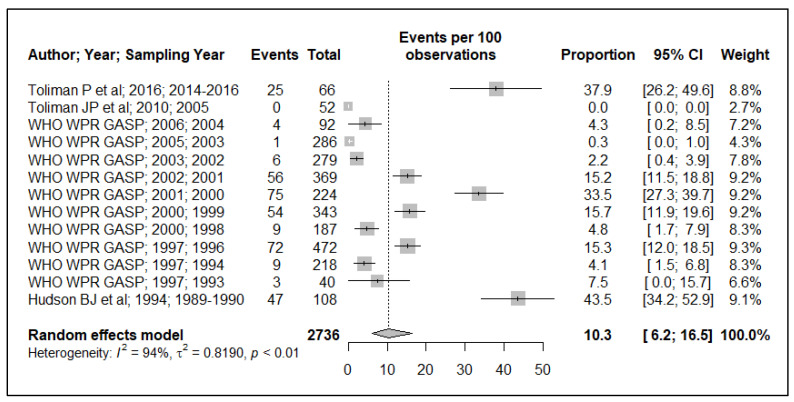
Tetracycline resistance prevalence estimate.

**Figure 5:**
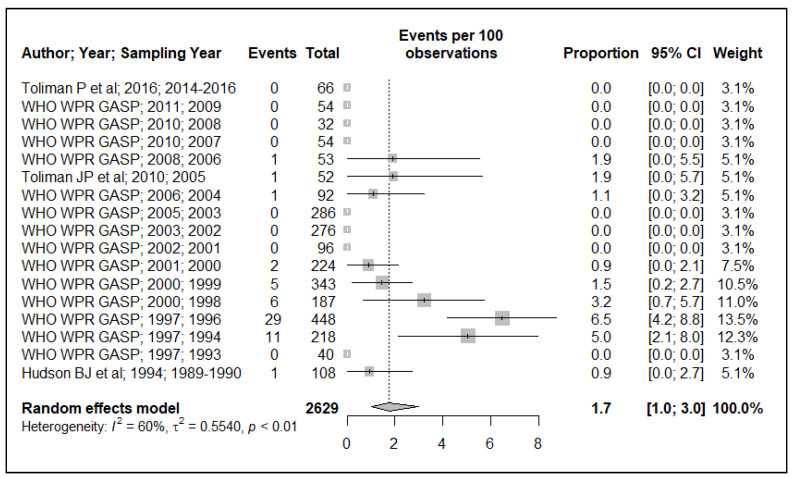
Quinolone (ciprofloxacin) resistance prevalence estimate.

**Figure 6:**
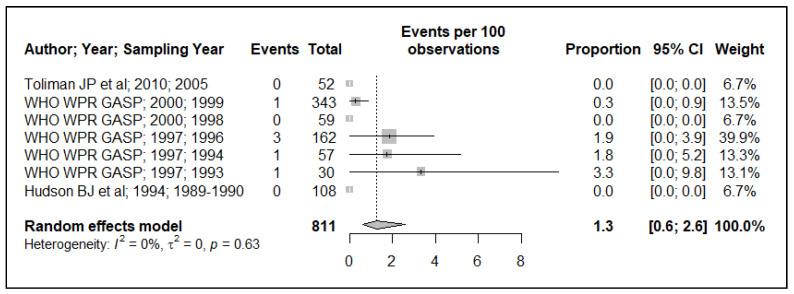
Spectinomycin resistance prevalence estimate.

**Figure 7:**
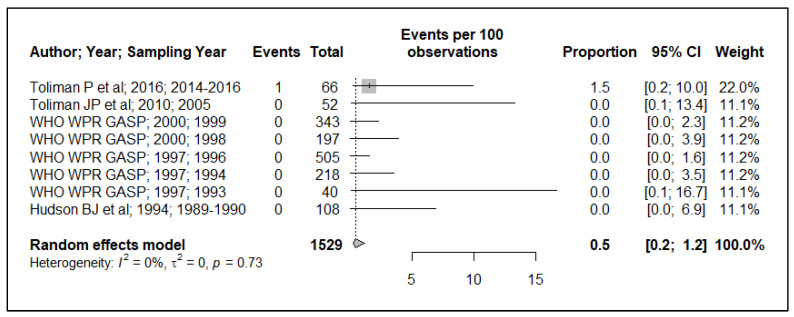
Cephalosporin resistance prevalence estimate.

Because of the small data points (based on year of sample collection) included in the meta-analysis, 17 data points for plasmid-mediated penicillin resistance (*n* = 17), chromosomally mediated penicillin resistance (*n* = 13), tetracycline resistance (*n* = 13), quinolone (ciprofloxacin) resistance (*n* = 17), aminoglycoside (spectinomycin) resistance (*n* = 7), macrolide (azithromycin) resistance (*n* = 2) and cephalosporin (ceftriaxone and or cefixime) resistance (*n* = 8), and, additionally, the small sample size used in the primary studies, these resulted in wide ninety-five percentage confidence intervals (95% CI) in this meta-analysis, see Figure 2, Figure 3, Figure 4, Figure 5, Figure 6 and Figure 7. Two studies that tested for azithromycin resistance reported zero cases (data not shown) [16,40].

### 3.1. Resistance Prevalence Estimates

#### 3.1.1. Penicillinase-Producing *N. gonorrhoeae* (PPNG)

The pooled PPNG resistance prevalence is estimated to be 37.9% (95% CI: 29.3–47.2) in this meta-analysis, see Figure 2. There was an increase in PPNG resistance prevalence, from 8.7% (95% CI: 5.6–13.9) in 1994 to as high as 74.1% (95% CI: 60.9–84) in 2007. A test of heterogeneity for PPNG prevalence showed that the reported prevalence was heterogeneous, I^2^ = 95% (*p* ≤ 0.05), see Figure 2.

#### 3.1.2. Chromosomally-Mediated Penicillin Resistance

The prevalence of chromosomally-mediated resistance to penicillin (CMR-penicillin) was significantly heterogeneous (I^2^ = 94%), see Figure 3. The pooled CMR-penicillin prevalence estimate is 4.3% (95% CI: 2–8.8). Despite the pooled prevalence being below the 5% threshold, it is important to know that five papers reported prevalence above the threshold, ranging from 6.2–41% between 1998 and 2008.

### 3.2. Tetracycline Resistance

Generally, there was an increase in the reported tetracycline resistance prevalence over time. The pooled tetracycline resistance prevalence estimate is 10.3% (95% CI: 6.2–16.5%), just above the 5% threshold, see Figure 4. However, interpreting this must be made with caution, given the heterogeneity (I^2^ = 93%) of the reported prevalence, with the mention of a high reported resistance prevalence in a recent study in 2016, 37.9% (95% CI: 26.2–49.6).

### 3.3. Quinolone Resistance

Overall, the reported quinolone resistance prevalence in the primary studies was heterogeneous (I^2^ = 60%), see Figure 5. The pooled prevalence estimate is below the 5% threshold, 1.7% (95% CI: 1–3). There was an observed decline in ciprofloxacin resistance prevalence beginning in late 1999. However, given the wide 95% CI observed for individual studies in this meta-analysis, the fall in quinolone resistance prevalence could not be ascertained.

### 3.4. Aminoglycoside Resistance

The reported aminoglycoside (spectinomycin) resistance prevalence is homogeneous (I^2^ = 0.0%). The estimated pooled prevalence is below the threshold, 1.3% (95% CI: 0.6–2.6), see Figure 6. Despite the homogeneity of the reported prevalence in this meta-analysis, the 95% CI values for individual studies are large. Thus, the interpretation of this estimate must be cautiously made.

### 3.5. Cephalosporin Resistance

The reported resistance prevalence from the papers analyzed for cephalosporin resistance was homogenous (I^2^ = 0.0%), with an estimated prevalence of 0.5% (95% CI: 0.2–1.2), see Figure 7. Because there were few data points and a small sample size employed in the primary studies, this resulted in a wide 95% CI. Therefore, caution is also needed in the interpretation of this estimate.

## 4. Discussion

In this prevalence meta-analysis, the pooled estimates of resistance prevalence in the last 30 years for PNG are under the 5% threshold for most antibiotics except for PPNG (37.9%) and tetracycline (10.3%). Yet, interpretations of these pooled estimates must be made with caution because resistance prevalence above the threshold has been reported. Most of the PNGs’ NG AMR data were from the WHO WPR GASP. During PNGs participation in the WPR GASP, PNG consistently submitted samples to the NG AMR surveillance program between 1993 and 2009. One-hundred and ninety-three (193) samples were submitted on average per year by PNG to the program, similar to Brunei, Japan, Korea, the Philippines, Vanuatu, and Vietnam. However, this was lower than Australia, Hong Kong, and Fiji, who were submitting 600–3000 samples per year in that same period. The main limitation of PNG NG AMR data was that the samples were collected from a few sites, and restricted to STI clinics.

Our prevalence meta-analysis showed an increase in PPNG prevalence from 1989 to 2009. PNG has reported some of the highest prevalence of PPNG in the WHO WPR and SEAR. The pooled PPNG prevalence estimate (37.9%, 95% CI: 29.3–47.2) in this meta-analysis is similar to that observed in other countries in the WHO WPR and SEAR, such as Hong Kong (32.4%), Malaysia (30%), and Vietnam (32.5%) [41]. Despite high PPNG prevalence in PNG, treatment with augmentin (amoxicillin-clavulanic acid, a penicillinase inhibitor) is used effectively in treating PPNG-positive NG strains. However, of importance to note is that a resistance prevalence of 6.1% towards amoxicillin-clavulanic acid was recently reported [40].

Japan has reported the highest prevalence of CMR to penicillin (39%), whereas other countries in the WPR and SEAR reported CMR to penicillin in the range of 10–20% [41]. The pooled estimate for CMR penicillin in the current study for PNG is one of the lowest (4.3%) in the WHO WPR and SEAR, other than Fiji (3.6%) [41]. However, interpreting it must be done with caution because of the small number of studies and sample sizes used to calculate the pooled prevalence estimate, and the fact that five studies used in our calculations reported prevalence above the 5% threshold, in the range of 6–41% (see Figure 3). 

Tetracycline is not the recommended antibiotic for treating gonorrhea, but is sporadically used. Our analysis showed an increase in tetracycline resistance prevalence between 1989 and 2000, followed by a decline between 2001 and 2004 in PNG. Surprisingly, a spike in prevalence was reported in 2016 in PNG, estimated at 37.6% (95% CI: 26.2–49.6), see Figure 4. The recently reported tetracycline resistance prevalence in PNG in 2016 [40] is much higher when compared to that of Australia in 2017 (12–21%) [42].

Several studies in PNG have reported a high prevalence of ciprofloxacin resistance in the mid to late 1990s, followed by a decline. A similar trend was seen for spectinomycin resistance. The declining resistance prevalence may be due to the strict use of amoxicillin and azithromycin. With the rise of ciprofloxacin resistance in the WPR (6–15% in New Caledonia; 16–30% in urban Australia; and 71–100% in China, Philippines, and Vietnam), PNG needs research and surveillance to ascertain the observed decline [43].

The only three countries in the WHO WPR that are still using penicillin and azithromycin for treating gonorrhea are PNG, Fiji, and the Solomon Islands. The rest of the countries in the region use cephalosporins (ceftriaxone or cefixime) and azithromycin [41,44]. However, PNG has a new treatment guideline that recommended the change of treatment to cephalosporin (ceftriaxone and cefixime) and azithromycin [18]. Though this change in treatment represents an important step towards consistency in treatment strategies for gonorrhea in the WPR and SEAR, there were insufficient NG AMR data in PNG to inform this change [18].

Our analysis showed that the estimated pooled resistance prevalence for most antibiotics is below the 5% threshold. This suggested the possibility to use other drug combinations, then to quickly use the last line of treatment. At the same time, it must be noted that most of the studies included in this meta-analysis were conducted a decade ago. Thus, this could mean that the estimates reported here may be outdated due to the lack of recent NG AMR surveillance in PNG.

The lack of representative and updated NG AMR data in PNG emphasizes the need to explore methods other than the culture method, such as molecular methods, to complement and support the culture method for NG AMR surveillance. Molecular testing has increasingly been used for NG and NG AMR testing in many countries, and is proving to be an invaluable tool for NG AMR surveillance [10]. Given its advantages discussed earlier, its adoption for use in PNG would be of great value to enhance NG AMR surveillance.

### Limitations

We acknowledge that there are limitations to this study. First, the article search was done in the MEDLINE, EMBASE, Google Scholar, and Papua New Guinea Institute of Medical Research Library databases; therefore, articles not indexed in these databases may have been missed. Second, the *N. gonorrhea* AMR prevalence results presented here are as reported in the primary studies, where multiple drug resistance by individual samples were not reported. Therefore, it is not possible to determine the presence of multi-drug resistant *N. gonorrhea* strains from these articles. Third, the data points analyzed in this study are small (15 papers and reports, covering 17 data points based on year of sample collection). The small data points and the small sample size analyzed in the primary studies have resulted in large 95% CI values in this analysis. Therefore, caution is required in the interpretation of these estimates until additional, more robust data is available.

## 5. Conclusions

In Papua New Guinea (PNG), penicillin and azithromycin remain the standard treatment for gonorrhea. That may soon change as per the newly drafted National STI Treatment Guidelines. Cephalosporins (cefixime and ceftriaxone) and azithromycin are now the recommended treatment for NG in PNG. However, PNG does not have sufficient NG AMR data to inform this change, which was acknowledged in the new treatment guidelines. This highlights the need for evidence-based research and surveillance of NG AMR in PNG.

In support of the latter, we showed that PNG has no national representative and updated NG AMR data in the last decade. Moreover, our meta-analysis showed inconsistencies in the reported NG AMR prevalence.

Though the culture method remains the “gold standard” for NG AMR testing, it requires viable NG bacteria. Maintaining viable bacteria for culture is often challenging in PNG because of the logistical difficulties and costs associated with the storage and transportation of viable NG bacteria, which severely affects NG AMR testing. Thus, it is appropriate to consider molecular testing methods for NG AMR testing and surveillance in PNG. Considering the advantages discussed earlier, molecular methods could be established easily, and used to enhance NG AMR surveillance quickly and efficiently in PNG.

## Data Availability

The datasets used and/or analyzed for the current study are available from the corresponding author on reasonable request.

## References

[1] Whiley M.D., Goire N., Lahra M.M., Donovan B., Limnios E.A., Nissen D.M., Sloots P.T. (2012). The ticking time bomb: Escalating antibiotic resistance in *Neisseria gonorrhoeae* is a public health disaster in waiting. J. Antimicrob. Chemother..

[2] Unemo M., Shafer M.W. (2014). Antimicrobial Resistance in *Neisseria gonorrhoeae* in the 21st Century: Past, Evolution, and Future. Clin. Microbiol. Rev..

[3] Młynarczyk-Bonikowska B., Majewska A., Malejczyk M., Młynarczyk G., Majewski S. (2020). Multiresistant *Neisseria gonorrhoeae*: A new threat in second decade of the XXI century. Med. Microbiol. Immunol..

[4] Whiley D.M., Lahra M.M., Unemo M. (2015). Prospects of untreatable gonorrhea and ways forward. Future Microbiol..

[5] World Health Organization (2012). Global Action Plan to Control the Spread and Impact of Antimicrobial Resistance in Neisseria Gonorrhoeae. https://apps.who.int/iris/handle/10665/44863.

[6] WHO (2015). Global Action Plan on Antimicrobial Resistance. http://www.who.int/antimicrobial-resistance/publications/global-action-plan/en/.

[7] Tapsal J. (2001). Antimicrobial Resistance in Neisseria Gonorrhoeae.

[8] Unemo M., Ballard R., Ison C., Lewis L., Ndowa F., Peeling R., WHO (2013). Laboratory Diagnosis of Sexually Transmitted Infections, Including Human Immunodeficiency Virus.

[9] WHO (2016). Guidelines for the Treatment of *Neisseria gonorrhoeae*. https://apps.who.int/iris/bitstream/handle/10665/246114/9789241549691-eng.pdf;jsessionid=CDF3A406C819BC7FBA92DFB2F48DB6D9?sequence=1.

[10] Whiley D.M., Trembizki E., Buckley C., Freeman K., Baird R.W., Beaman M., Chen M., Donovan B., Kundu R.L., Fairley C.K. (2017). Molecular Antimicrobial Resistance Surveillance for *Neisseria gonorrhoeae*, Northern Territory, Australia. Emerg. Infect. Dis..

[11] WHO (2018). Review of National Treatment Guidelines for Sexually Transmitted Infections in the Western Pacific Region.

[12] Unemo M., Lahra M.M., Cole M., Galarza P., Ndowa F., Martin I., Dillon J.R., Ramon-Pardo P., Bolan G., Wi T. (2019). World Health Organization Global Gonococcal Antimicrobial Surveillance Program (WHO GASP): Review of new data and evidence to inform international collaborative actions and research efforts. Sex. Health.

[13] Nishijima T., Nand D., David N., Bauri M., Carney R., Htin K.C.W., Shwe Y.Y., Gurung A., Mahiane G., Ishikawa N. (2020). Prevalence of syphilis, gonorrhoea and chlamydia in women in Fiji, the Federated States of Micronesia, Papua New Guinea and Samoa, 1995–2017: Spectrum-STI model estimates. West. Pac. Surveill. Response J..

[14] Kelly-Hanku A., Willie B., Weikum D., Boli Neo R., Kupul M., Coy K., Hou P., Aeno H., Ase S., Gabuzzi J. (2018). Kauntim mi tu: Multi-Site Summary Report from the Key Population Integrated Bio-Behavioural Survey, Papua New Guinea.

[15] Vallely A., Page A., Shannon Dias S., Siba P., Lupiwa T., Law G., Millan J., Wilson P.D., Murray M.J., Toole M. (2010). The Prevalence of Sexually Transmitted Infections in Papua New Guinea: A Systematic Review and Meta-Analysis. PLoS ONE.

[16] Toliman J.P., Lupiwa T., Law J.G., Reeder C.J., Siba M.P. (2010). *Neisseria gonorrhoeae* isolates from four centres in Papua New Guinea remain susceptible to amoxycillin-clavulanate therapy. PNG Med. J..

[17] NDoH, P.N.G. National Department of Health (2012). Standard Treatment Guidelines For Common Illness of Adults in Papua New Guinea. A Manual for Nurses, Health Extension Officers and Doctors, National Department of Health.

[18] NDoH (2019). Papua New Guinea Standard Management of Sexually Transmitted Infections and Genital Conditions.

[19] PRISMA Transparent Reporting of Systematic Reviews and Meta-Analyses. http://www.prisma-statement.org.

[20] National Library of Medicine (2020). National Library of Medicine. National Center for Biotechnology Information. https://pubmed.ncbi.nlm.nih.gov.

[21] EMBASE (2020). Cited. https://www.embase.com/#search.

[22] The EndNote Team (2013). EndNote.

[23] R Core Team (2017). R: A Language and Environment for Statistical Computing.

[24] Viechtbauer W. (2010). Conducting Meta-Analyses in R with the metafor Package. J. Stat. Softw..

[25] Barendregt J.J., Doi S.A., Lee Y.Y., Norman R.E., Vos T. (2013). Meta-analysis of prevalence. J. Epidemiol. Community Health.

[26] Riley R.D., Higgins J.P., Deeks J.J. (2011). Interpretation of random effects meta-analyses. BMJ.

[27] WHO Western Pacific Region Gonococcal Antimicrobial Surveillance Programme (1997). Surveillance of Antibiotic Susceptibility of *Neisseria gonorrhoeae* in the WHO Western Pacific region 1992-4. Genitourin. Med..

[28] The WHO Western Pacific Region Gonococcal Antimicrobial Surveillance Programme (1997). Antimicrobial resistance in gonococci, WHO Western Pacific Region, 1996. Commun. Dis. Intell..

[29] The WHO Western Pacific Gonococcal Antimicrobial Surveillance Programme (2000). Surveillance of antibiotic resistance in *Neisseria gonorrhoeae* in the WHO Western Pacific Region, 1998. Commun. Dis. Intell..

[30] The WHO Western Pacific Region Gonococcal Antimicrobial Surveillance Programme (2000). Surveillance of antibiotic resistance in *Neisseria gonorrhoeae* in the WHO Western Pacific Region, 1999. Commun. Dis. Intell..

[31] The WHO Western Pacific Gonococcal antimicrobial Surveillance Programme (2001). Surveillance of antibiotic resistance in *Neisseria gonorrhoeae* in the WHO Western Pacific Region, 2000. Commun. Dis. Intell..

[32] The WHO Western Pacific Gonococcal Antimicrobial Surveillance Programme (2002). Surveillance of antibiotic resistance in *Neisseria gonorrhoeae* in the WHO Western Pacific Region, 2001. Commun. Dis. Intell..

[33] WHO Western Pacific Gonococcal Antimicrobial Surveillance Programme (2003). Surveillance of antibiotic resistance in *Neisseria gonorrhoeae* in the WHO Western Pacific Region, 2002. Commun. Dis. Intell..

[34] The WHO Western Pacific Gonococcal Antimicrobial Surveillance Programme (2005). Surveillance of antibiotic resistance in *Neisseria gonorrhoeae* in the World Health Organization Western Pacific Region, 2003. Commun. Dis. Intell..

[35] The WHO Western Pacific Gonococcal Antimicrobial Surveillance Programme (2006). Surveillance of antibiotic resistance in *Neisseria gonorrhoeae* in the WHO Western Pacific Region, 2004. Commun. Dis. Intell..

[36] The WHO Western Pacific Gonococcal Antimicrobial Surveillance Programme (2008). Surveillance of Antibiotic Resistance in *Neisseria gonorrhoeae* in The Who Western Pacific Region, 2006. Commun. Dis. Intell..

[37] The WHO Western Pacific and South East Asian Gonococcal Antimicrobial Surveillance Programmes (2010). Surveillance of antibiotic resistance in *Neisseria gonorrhoeae* in the WHO Western Pacific and South East Asian Regions, 2007–2008. Commun. Dis. Intell..

[38] The WHO Western Pacific and South East Asian Gonococcal Antimicrobial Surveillance Programmes (2011). Surveillance of antibiotic resistance in *Neisseria gonorrhoeae* in the WHO Western Pacific and South East Asian Regions, 2009. Commun. Dis. Intell..

[39] Hudson B.J., van der Meijden W.I., Lupiwa T., Howard P., Tabua T., Tapsall J.W., Phillips E.A., Lennox V.A., Backhouse J.L., Pyakalyia T. (1994). A survey of sexually transmitted diseases in five STD clinics in Papua New Guinea. Papua New Guin. Med. J..

[40] Toliman P., Yoannes M., Toto B., Koata A. (2016). Gonococcal Antimicrobial Susceptibility Survey Goroka, Eastern Highlands Province.

[41] The WHO Western Pacific and South East Asian Gonococcal Antimicrobial Surveillance Programmes (2012). Surveillance of Antibiotic Resistance in Neisseria Gonorrhoeae in The Who Western Pacific And South East Asian Regions, 2010. Commun. Dis. Intell..

[42] Lahra M.M., Enriquez R., Robert George C.R.R. (2018). Australian Gonococcal Surveillance Programme Annual Report, 2017. Commun. Dis. Intell..

[43] Arya R., Antonisamy B., Kumar S. (2012). Sample size estimation in prevalence studies. Indian J. Pediatrics.

[44] WHO (2017). Gonococcal Antimicrobial Resistance in the Western Pacific Region. https://apps.who.int/iris/rest/bitstreams/1148004/retrieve.

